# A Review of Software and Mobile Apps to Support the Clinical Diagnosis of Hansen Disease

**DOI:** 10.2196/47142

**Published:** 2023-08-18

**Authors:** Wilbert Dener Lemos Costa, Alan Maicon de Oliveira, Guilherme José Aguilar, Luana Michelly Aparecida Costa dos Santos, Luiz Ricardo Albano dos Santos, Dantony de Castro Barros Donato, Felipe Foresto, Marco Andrey Cipriani Frade

**Affiliations:** 1 Intersection LTDA Ribeirão Preto Brazil; 2 Ribeirão Preto Medical School University of São Paulo Ribeirão Preto Brazil; 3 School of Pharmaceutical Sciences of Ribeirão Preto University of São Paulo Ribeirão Preto Brazil; 4 Faculty of Philosophy, Sciences and Letters at Ribeirão Preto University of São Paulo Ribeirão Preto Brazil; 5 School of Economics, Business Administration and Accounting at Ribeirão Preto University of São Paulo Ribeirão Preto Brazil

**Keywords:** software, mobile apps, leprosy, medical informatics, Mycobacterium leprae, clinical diagnosis, Hansen disease, mHealth, mobile health, mobile app, Hansen, dermatology, scoping review, skin, diagnosis, diagnostic

## Abstract

This scoping review indicates a lack of scientific articles that specifically explore software and mobile applications designed to assist in the clinical diagnosis of leprosy, and our findings have provided insights into the available tools, their usage methods, and the benefits offered by health technologies.

## Introduction

Hansen disease, or leprosy, is a chronic infectious disease caused by *Mycobacterium leprae* (*M leprae*). It mainly affects the skin’s superficial nerves and peripheral nerve trunks and can also impact the eyes and internal organs. If untreated, leprosy becomes contagious and can lead to physical disabilities. Additionally, it imposes significant social, emotional, and economic burdens [1].

The diagnosis of leprosy is based on assessing clinical presentation, including signs and symptoms. Leprosy cases are classified into two types for treatment: paucibacillary and multibacillary. Paucibacillary cases have 1 to 5 skin lesions and no bacilli in a bacilloscopy, whereas multibacillary cases have more than 5 skin lesions and/or the presence of bacilli in a bacilloscopy [1,2].

The World Health Organization (WHO) encourages early leprosy detection and supports the development of mobile health (mHealth) innovations for this purpose [2]. The use of computational tools in health care is expanding, providing health care professionals with enhanced agility and precision and improving the overall patient-physician experience [3,4].

This study aimed to identify the scientific literature on software and mobile apps designed to assist in the clinical diagnosis of leprosy and describe their main characteristics.

## Methods

We used the methodology developed by Arksey and O’Malley [5], following the PRISMA-ScR (Preferred Reporting Items for Systematic Reviews and Meta-Analyses Extension for Scoping Reviews) checklist. This included defining the eligibility criteria, devising a search strategy (Multimedia Appendix 1), selecting sources of evidence (PubMed and Embase), collecting data, and synthesizing results. All steps of the methodology were documented in a previously registered protocol [6].

## Results

### Selection of Studies

**Figure 1:**
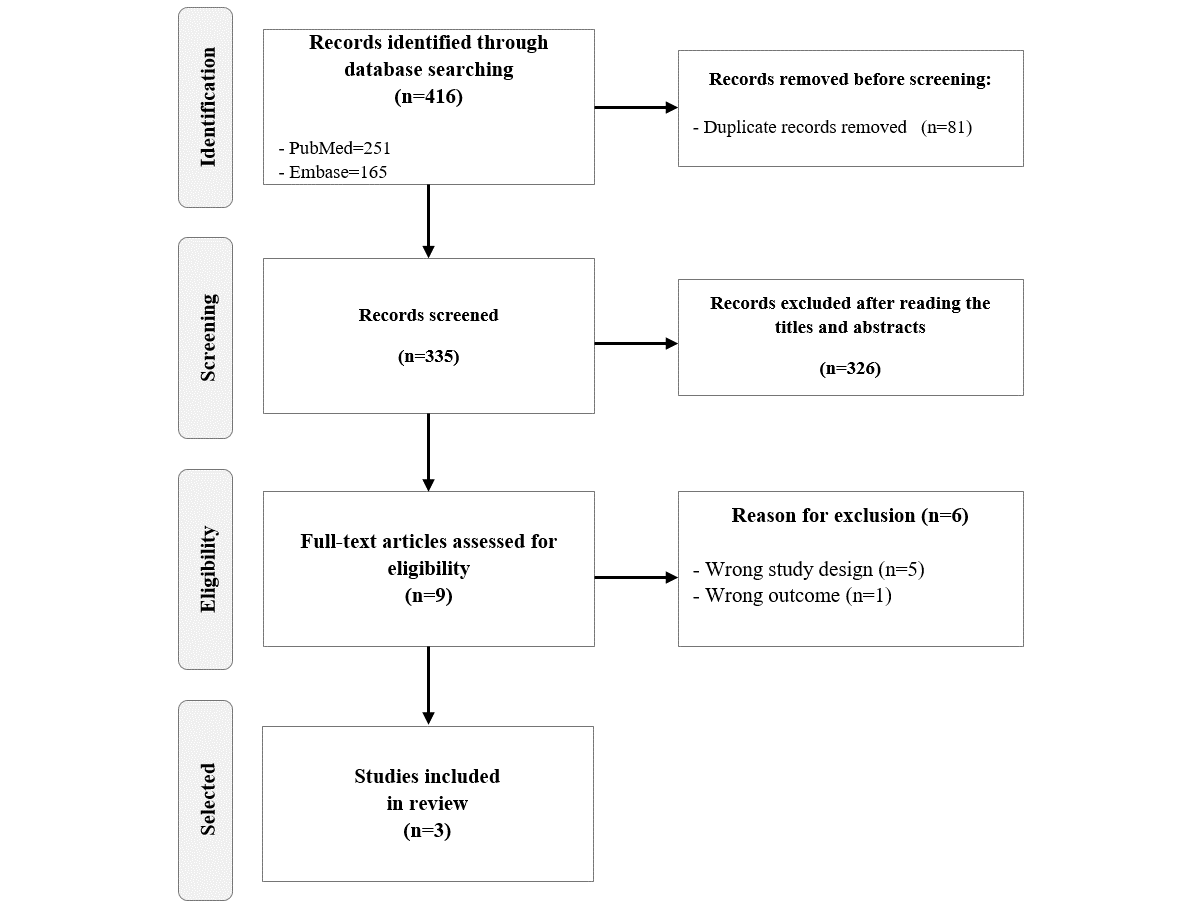
Flowchart outlining the study selection and inclusion process.

In step 1, a database search yielded 416 publications. After removing duplicates (n=81), step 2 involved an eligibility assessment based on title and abstract analysis. Step 3 included reading the full texts of the selected studies. Excluded articles were mainly protocols or conference abstracts. Ultimately, 3 publications were analyzed in this scoping review [7-9] (Figure 1).

### Characteristics of the Included Studies

The studies included in this review were published between 2018 and 2021 (Table 1). Two were initiatives conducted in Brazil [8,9], and one was from the not-for-profit organization Netherlands Leprosy Relief [7]. One of the studies analyzed [8] used a computerized method to assess the Mitsuda test. This test involves assessing the skin’s immune response and can aid in identifying individuals at risk of developing illness upon exposure to *M leprae*. SkinApp, as described by Mieras et al [7], was still in development and was undergoing updates based on applicability tests, despite having already undergone several development stages. De Souza et al [9] proposed a cross-platform app, comprising a vast database to assist in the screening and differentiation of leprosy types. The Brazilian database Information System for Notifiable Diseases was used to develop this app.

**Table 1:** Aims and outcomes of the included studies.

Reference and year of publication	Study aims	Software/app	Methodology used by the software/app	Positive aspects	Negative aspects
Mieras et al [7], 2018	To describe the development process of a mobile phone app that supports peripheral health workers in diagnosing and treating skin diseases in resource-poor settings	SkinApp	Algorithm to support the process of diagnosis Descriptions of skin diseases, supporting photos, as well as treatment and referral advice	Training tool Easy to use Clear treatment advice (ie, narrative and illustrative content was considered clear) Clinical validation of a patient with a skin disease Available in English and Portuguese (Android, Google Play Store; iOS, Apple App Store) Free of charge Can be used offline	Needs to improve intelligibility; a glossary explaining dermatological terminology could help A reporting option was also mentioned as a possible improvement Not all treatment options may be available The studies that were carried out did not address the performance of SkinApp as a diagnostic tool
Alecrim et al [8], 2019	To compare the results between the standardized reading and the total area of the Mitsuda test obtained by a computerized method that was structured by associating the digital dermatoscopy, the Dermatology Web system, and the Image Tool 3.0 software	Dermatology Web + Image Tool 3.0	Dermatology Web: photographic documentation of dermatological treatments and photo storage Image Tool 3.0: view, edit, analyze, process, save, and print images	Dermatology Web: can be used on any mobile platform or computer connected to the internet; ensured security and confidentiality of data stored in medical records Image Tool 3.0: area calculation; image calibrated in millimeters; delineation of the contours of the reaction; results in a total area in square millimeters Dermatology Web + Image Tool 3.0: improves reading precision; allows for the computerization of records	Dermatology Web + Image Tool 3.0: functions are not centralized in a single software
De Souza et al [9], 2021	To develop a cross-platform app for leprosy screening based on artificial intelligence	App for leprosy screening	Supervised learning (random forest)	Improves coverage and scalability to the health service regarding the choice of an appropriate treatment for leprosy Accessibility via mobile or desktop option Speed, scalability, and broadcasting to fight leprosy without compromising accuracy High accuracy (92.38%), sensitivity (93.97%), and specificity (87.09%)	Not available without an internet connection Quality of the data used by the app depends on many factors (quality of the items requested by the forms and their correct interpretation, correct clinical assessment of the patient, proper filling out of the forms)

## Discussion

### Principal Findings

This review indicates a scarcity of software and mobile apps specifically designed to assist in the clinical diagnosis of leprosy, with their development documented in scientific articles. Despite their promising attributes for clinical practice, it is advisable to test these technologies using controlled trials to determine their actual impact.

The Global Leprosy Strategy 2021-2030 [2], initiated by the WHO, emphasized the importance of developing eHealth innovations to improve the diagnosis and care of patients with leprosy. Others have also supported the potential of digital technologies in health care [3,4]. As a result, our study aligns with the WHO initiative and offers valuable insights for enhancing strategies in this domain.

In 2020, a total of 127,396 new cases of leprosy were reported worldwide. As a result, Brazil ranks second globally in terms of leprosy cases, with India having the highest number of cases [2]. These data may help explain why the majority of the software and apps described in our study was developed in Brazil.

It is important to note that not all health technology tools have their development documented in scientific studies [10], and it is possible that relevant evidence might not have been indexed in the databases we used for our search. Consequently, some initiatives [10] did not meet our inclusion criteria. Nevertheless, our study underscores the importance of documenting technological advancements in scientific studies and encourages their implementation through controlled trials.

### Limitations

Our study involved searching for relevant studies using 2 databases. We did not use additional health databases or multidisciplinary databases, which may have influenced our results. Furthermore, we specifically focused on publications related to the clinical diagnosis of leprosy, excluding studies pertaining to laboratory diagnosis and disease follow-up. As a result, the scope of our findings was limited.

## Appendix

Multimedia Appendix 1 Search strategy for databases.

Multimedia Appendix 2 PRISMA-ScR (Preferred Reporting Items for Systematic Reviews and Meta-Analyses Extension for Scoping Reviews) checklist.

## Abbreviations

mHealth: mobile health
PRISMA-ScR: Preferred Reporting Items for Systematic Reviews and Meta-Analyses Extension for Scoping Reviews
WHO: World Health Organization
