# Supplementary material for: A Review of Software and Mobile Apps to Support the Clinical Diagnosis of Hansen Disease
Source: JMIR Dermatol. 2023 Aug 18;6:e47142. doi: 10.2196/47142 (PMC10474501; doi:10.2196/47142)
Supplement: Multimedia Appendix 1 [file derma_v6i1e47142_app1.docx]

**SUPPLEMENTARY INFORMATION**

**Supplementary material 1 -** Search strategy adapted to databases

1. **PUBMED**

|  | **Search strategy** | | **Results** | |
| --- | --- | --- | --- | --- |
| **#1** |  | "Leprosy"[mesh] OR "Leprosy"[tiab] OR "Hansen's Disease"[tiab] OR "Hansen Disease"[tiab] | 27,134 |  |
| **#2** |  | "Software"[mesh] OR "Software"[tiab] OR "Mobile Applications"[mesh] OR "Mobile Applications"[tiab] OR "Mobile"[tiab] OR "Applications"[tiab] OR "Application"[tiab] OR "Medical Informatics"[mesh] OR "Health informatics"[tiab] OR "App"[tiab] OR "Apps"[tiab] | 2,342,938 |  |
| **#3** |  | "Diagnosis"[mesh] OR "Clinical Diagnosis"[tiab] OR "Diagnostic Imaging"[tiab] OR "Diagnostic"[tiab] OR "Identification"[tiab] OR "Triage"[tiab] OR "Screening"[tiab] OR "Assessment"[tiab] | 11,195,519 |  |
| **#4** |  | Search: (#1 AND #2 AND #3) | 251 |  |

1. **EMBASE**

|  | **Search strategy** | | **Results** | |
| --- | --- | --- | --- | --- |
| **#1** |  | "Leprosy":kw OR "Leprosy":ab,ti OR "Hansens Disease":ab,ti OR "Hansen Disease":ab,ti | 26,467 |  |
| **#2** |  | "Software":kw OR "Software":ab,ti OR "Mobile Applications":kw OR "Mobile Applications":ab,ti OR "Mobile":ab,ti OR "Applications":ab,ti OR "Application":ab,ti OR "Medical Informatics":kw OR "Health informatics":ab,ti OR "App":ab,ti OR "Apps":ab,ti | 2,233,115 |  |
| **#3** |  | "Diagnosis":kw OR "Clinical Diagnosis":ab,ti OR "Diagnostic Imaging":ab,ti OR "Diagnostic":ab,ti OR "Identification":ab,ti OR "Triage":ab,ti OR "Screening":ab,ti OR "Assessment":ab,ti | 4,422,864 |  |
| **#4** |  | Search: #1 AND #2 AND #3 | 165 |  |
